# Succinate-GPR91 signaling promotes cardiomyocyte metabolic reprogramming and NAD^+^ production to alleviate HFpEF

**DOI:** 10.21203/rs.3.rs-7556256/v1

**Published:** 2025-09-17

**Authors:** YuMeng Jia, WenHui Niu, Lu Liu, Qun Zhang, DingWei Li, TangYu Dai, Jurgen Wess, Lei Wang, Jie Du

**Affiliations:** Capital Medical University; Capital Medical University; Capital Medical University; Capital Medical University; Capital Medical University; Capital Medical University; National Institute of Diabetes and Digestive and Kidney Diseases; Capital Medical University; Beijing Anzhen Hospital, Capital Medical University

**Keywords:** Succinate, GPR91, HFpEF, Cardiomyocyte, AMPK, NAD+

## Abstract

**Background:**

Disrupted cardiomyocyte energy metabolism is a hallmark of heart failure with preserved ejection fraction (HFpEF). Succinate, a key intermediate of the tricarboxylic acid cycle, is markedly decreased in HFpEF myocardium. Beyond its metabolic role, succinate functions as a signaling molecule that activates GPR91 to regulate metabolic and immune pathways. However, the precise contributions and mechanisms of cardiomyocyte succinate–GPR91 signaling in HFpEF pathogenesis remain largely unknown.

**Methods:**

HFpEF models were established in wild-type, global GPR91 knockout, and cardiomyocyte-specific GPR91 knockout mice with or without succinate supplementation. Cardiac structure, function, and metabolic phenotypes were assessed using echocardiography, histology, and molecular assays. Transcriptome sequencing of myocardial tissues was performed to identify succinate–GPR91–dependent signaling pathways. Mechanistic studies in isolated cardiomyocytes were conducted to validate pathway regulation and clarify downstream molecular mechanisms. Rescue experiments were further carried out to confirm the functional relevance of succinate–GPR91 signaling in cardiomyocyte metabolism and HFpEF progression.

**Results:**

Cardiac succinate levels and GPR91 expression were markedly decreased in HFpEF mice. Succinate supplementation restored systemic metabolism, improved diastolic function, and attenuated myocardial hypertrophy and fibrosis in wild-type (WT) HFpEF mice, but these protective effects were lost in both global Gpr91^−/−^ and cardiomyocyte-specific Gpr91^ΔCM^ knockouts. Transcriptomic analysis demonstrated that succinate activated AMPK signaling and enriched pathways related to glucose–lipid metabolism and NAD^+^ biosynthesis in Gpr91^fl/fl^ but not in Gpr91^ΔCM^ hearts. Mechanistically, succinate enhanced AMPK phosphorylation and NAD^+^ production via Gq-mediated signaling, thereby promoting metabolic reprogramming.

**Conclusion:**

These findings identify the succinate–GPR91 axis as a critical regulator of cardiometabolic homeostasis and a potential therapeutic target in HFpEF.

## Background

Heart failure with preserved ejection fraction (HFpEF) has become the predominant subtype of heart failure, accounting for more than half of all cases worldwide, yet effective therapeutic strategies remain lacking [1, 2]. Increasing evidence suggests that HFpEF is a metabolic disorder–driven syndrome, strongly associated with comorbidities such as obesity, hypertension, type 2 diabetes, and dyslipidemia [3–5]. At the cellular level, cardiomyocyte energy metabolism dysfunction constitutes a central pathophysiological mechanism, largely attributed to mitochondrial impairment and leading to reduced ATP generation[6–10]. Transcriptomic profiling revealed significantly impaired metabolism pathways, including the tricarboxylic acid (TCA) cycle, electron transport chain, and oxidative phosphorylation et al.[11, 12]. Myocardium metabolomic analyses further demonstrated a consistent reduction in succinate, a key intermediate of the TCA cycle, in murine and human HFpEF heart tissues [11, 13]. Beyond its role as a metabolic intermediate, succinate acts as a signaling molecule by activating its receptor, GPR91 (also known as SUCNR1), to regulate glucose–lipid metabolism, inflammatory responses, and cellular energy homeostasis[14–17]. Dysregulated succinate–GPR91 signaling has been implicated in obesity[18–22], hypertension[23, 24], diabetes, atherosclerosis, myocardial ischemia, arterial fibrillation, cardiac hypertrophy, metabolic dysfunction-associated steatohepatitis, non-alcoholic fatty liver disease, chronic neuroinflammation, rheumatoid arthritis et al. by regulating mitochondrial metabolism, inflammation, and fibrosis etal.[25–28] However, the precise role and molecular mechanisms of the succinate–GPR91 axis in HFpEF remain elusive.

AMP-activated protein kinase (AMPK) and nicotinamide adenine dinucleotide (NAD^+^) are central regulators of myocardial energy homeostasis. Reduced AMPK activity and NAD^+^ depletion are consistently observed in HFpEF, and pharmacological activation of either axis ameliorates metabolic dysfunction and adverse remodeling[29]. [30, 31]. Additionally, NAD^+^, a central cofactor in redox and energy metabolism, is significantly depleted in HFpEF, contributing to impaired energy balance[32, 33]. Notably, AMPK activation has been shown to enhance intracellular NAD^+^ levels, thereby improving metabolic function[34]. Yet, whether the succinate–GPR91 pathway regulates AMPK–NAD^+^ signaling in the HFpEF heart has not been determined.

In this study, we used a “two-hit” HFpEF mouse model to define the role of succinate–GPR91 signaling in cardiac metabolism. We demonstrate that succinate supplementation improves diastolic function, myocardial remodeling, and metabolic homeostasis in wild-type but not GPR91-deficient mice. Mechanistically, succinate activates Gq-dependent AMPK phosphorylation and enhances NAD^+^ biosynthesis, while nicotinamide partially rescues cardiac dysfunction in GPR91-deficient models. These findings identify succinate–GPR91 signaling as a critical regulator of AMPK–NAD^+^–mediated metabolic remodeling in HFpEF and a potential therapeutic target.

## Methods

### Experimental animals

C57BL/6J Gpr91^fl/fl^ mice were generated by GemPharmatech using CRISPR–Cas9 technology. Wild type, Myh6^Cre^ mice were purchased from GemPharmatech Co., Ltd, Strain No. T004713. Myh6-Cre Gpr91^fl/fl^ (GPR91^ΔCM^) mice were generated by crossing Gpr91^fl/fl^ and Myh6-Cre mice. Gpr91^−/−^ mice were purchased from Cyagen Biosciences. All the mice in the experiments were male only. All mice were housed with ad libitum access to food and water and maintained in a specific pathogen-free animal facility at an ambient temperature of 22°C and 50% humidity under a 12/12-h light–dark cycle (lights on at 06:00 and lights off at 18:00). Beginning at 8 weeks of age, mice were fed for 12 weeks with a regular chow diet or a high-fat diet (HFD) (Research Diet, Cat# D12492) plus Nω-Nitro-L-arginine methyl ester hydrochloride (L-NAME) (Sigma, Cat# N575125G) dissolved in drinking water (0.5 g/L, pH = 7.4). 1.5% Succinate (Sigma, Cat# 224731-500g) or 40mM Nicotinamide (Sigma, Cat# N0636-100G) was delivered in drinking water. Body weight was measured every week, and compared at the starting timepoint to ensure that there is no systematic bias in the allocation of animals to different experimental conditions. Blood pressure was measured non-invasively using a BP-2000 Blood Pressure Analysis System (Visitech Systems, USA) via the tail-cuff method. Blood glucose levels were measured using a Yuwell 710 blood glucose meter (Yuwell, China). Fasting blood glucose was measured after a 12-hour overnight fast, while random blood glucose was assessed in the morning without prior fasting. Fat content was determined using micro computed tomography (Micro CT, SKYSCAN 1276, Bruker, Germany). Echocardiography was performed at 12 weeks after the indicated diet to assess cardiac function. Upon euthanasia, tissues were weighed and instantly frozen in liquid nitrogen. Blood was collected in Eppendorf tubes containing EDTA (Solarbio, Cat# E1170) as an anticoagulant and plasma was collected after 3,000 rpm for 10 min at 4°C. Dry lung weight was determined after 48 hours of incubation in an oven at 37°C.

All experimental protocols for the animal studies were approved by the Capital Medical University’s Experimental Animal Center (approval AEEI-2023-165) and were carried out in accordance with the National Institutes of Health, Guide for the Care and Use of Laboratory Animals.

### Cell culture

Human cardiomyocyte cell line AC16 (ATCC, CRL-3568) was cultured in DMEM (Corning, Cat# 10013127) supplemented with 10% fetal bovine serum (Corning, Cat#35015-CV) and 1% penicillin-streptomycin (Procell, Cat# PB180120). To examine the phosphorylation level of AMPK, AC16 cells were treated with high glucose 33 mM(Procell, Cat# PB180418) and 200μM palmitate (Kunchuang Biotechnology, Cat# KC002) for 48 h, followed by pretreatment with 1μM YM-254890 (FUJIFILM, Cat# 568580-02-9) for 1 h and subsequent exposure to 400μM succinate (Sigma, Cat# 224731-500g) for 5 min. For downstream gene expression analysis, AC16 cells were treated with HG+PA for 48 h, and then co-incubated with YM-254890 and succinate for an additional 24 h.

### Isolation of primary NMCMs

Hearts were isolated from WT and Gpr91^−/−^ neonatal mice after surface disinfection with 75% ethanol. Blood clots and connective tissue were removed, and the heart tissues were minced into small pieces. The minced tissues were digested in 3 mL of 0.04% collagenase II solution at 37 °C for 5 minutes. Enzymatic digestion was terminated by adding 3 mL of 10% fetal bovine serum (FBS, v/v), and the supernatant was collected and centrifuged to obtain the cell pellet. This digestion step was repeated six times to maximize cell yield.

The collected cell pellets from each digestion were resuspended by gentle pipetting and pooled together. After adjusting the final cell density to 1 × 10^6^ cells/mL, the cell suspension was diluted fivefold using high-glucose DMEM medium supplemented with 10% FBS (v/v), 1% penicillin–streptomycin (v/v), and 1% BrdU (v/v). Cells were then seeded into laminin-coated culture plates and incubated at 37 °C in a humidified incubator with 5% CO_2_.

### Exercise Exhaustion Test

Following three days of adaptive training, mice were subjected to an exercise exhaustion test using a rotarod apparatus (Zhongshi Technology, China). The test protocol started with a rotation speed of 5 rpm for 5 seconds, followed by 10 rpm for another 5 seconds, and then gradually accelerated to 20 rpm/min. Mice were allowed to run until they fell from the rod or reached the maximum duration of 10 minutes. The latency to fall (in seconds) was recorded as a measure of exercise endurance. Each mouse underwent three independent trials with consistent performance, and the average value was taken as the final result.

### Intraperitoneal Glucose Tolerance Test (IPGTT)

After 16 hours of fasting, mice were subjected to IPGTT by intraperitoneal injection of glucose (Sigma, Cat# G7528) at a dose of 1 g/kg body weight dissolved in sterile saline. Tail vein blood glucose levels (mmol/L) were measured using a glucometer (Yuwell, China) at 0, 15, 30, 60, 90〿and 120 minutes after glucose administration. Anesthesia was not used, and mice were returned to their home cages immediately after the test.

### Intraperitoneal Insulin Tolerance Test (IPITT)

After 4 hours of fasting, IPITT was performed by intraperitoneal injection of insulin 1 U/kg (Solarbio, Cat# I8830) diluted in sterile saline. Tail blood glucose levels (mmol/L) were measured using a glucometer (Yuwell 710) at 0 (before injection), 15, 30, 60, 90, and 120 minutes after insulin administration. Anesthesia was not required, and the mice were returned to their original cages immediately after the test.

### Echocardiography

Mice were anesthetized with 1–2% isoflurane in 98–99% oxygen. Transthoracic echocardiography was performed using high-frequency, high-resolution digital imaging systems (Vevo 3100 and Vevo F2, Fujifilm VisualSonics) equipped with a 30 MHz MS400 transducer. The parasternal long-axis view was obtained in B-mode to identify the maximum left ventricular (LV) length. LV systolic function was assessed by M-mode measurements of the LV internal diameter under two-dimensional guidance. Left ventricular ejection fraction (LVEF) and fractional shortening (FS) were calculated based on these M-mode images. Diastolic function was evaluated using Pulsed-Wave Doppler (PWD) and Tissue Doppler Imaging (TDI) modes. Transmitral early (E) wave peak velocity and early diastolic annular velocity (E′) were measured, and the E/E′ ratio was calculated to assess LV diastolic function. Speckle-tracking based strain analysis was performed on the parasternal long-axis B-mode images using Vevo Strain software (v5.7.1) to determine global longitudinal strain (GLS). All measurements were repeated at least three times per mouse. After completion of the examination, mice recovered from anesthesia without complications and were promptly returned to their home cages. Echocardiographic parameters for the different experimental groups are summarized in Table S1

### Histological Analysis

Mice were euthanized prior to tissue collection, and hearts were excised without the induction of diastolic cardiac arrest. Samples were fixed in 4% paraformaldehyde (Biosharp, Cat# BLS39A) overnight, followed by standard paraffin embedding procedures. Paraffin-embedded tissues were sectioned at a thickness of 5 μm for subsequent staining.

Masson’s trichrome staining was performed using a commercially available kit (Solarbio, Cat# G1340), while hematoxylin and eosin (H&E) staining was conducted with an H&E staining kit (Solarbio, Cat# G1120). The stained slides were digitized using a high-resolution whole-slide scanner (PANNORAMIC SCAN II, 3DHISTECH) and analyzed with CaseViewer software (3DHISTECH). Fibrosis quantification and morphometric assessments were performed using ImageJ software (NIH, USA).

For wheat germ agglutinin (WGA) staining, tissue sections were incubated with WGA (Thermo Fisher, Cat# W21405) diluted in PBS for 2 hours at room temperature. Nuclei were counterstained with anti-fade mounting medium containing DAPI (Beyotime, Cat# P0131). Fluorescence images were acquired using a fluorescence inverted microscope (Ti2-U, Nikon, Japan) and quantified using ImageJ.

Oil Red O staining was performed on cryosections prepared by fixing heart tissues in 4% paraformaldehyde for 4 hour immediately after excision, dehydrating in 30% sucrose solution until sinking, embedding in OCT compound, and cutting 8 μm sections. After equilibration to room temperature, sections were pretreated with 60% isopropanol for 3 minutes to preserve lipid solubility. Oil Red O working solution was prepared by mixing Oil Red O stock solution (Solarbio, Cat# G1261) with deionized water at a 2:3 volume ratio and filtering through filter paper. Sections were stained with the filtered solution for 30 minutes, rinsed quickly with 60% isopropanol to remove nonspecific staining, and gently washed with PBS. Nuclei were counterstained with hematoxylin for 1 minute, rinsed under running water, and mounted with neutral resin (Beyotime, Cat# C0173-100ml). Images were captured using the same fluorescence inverted microscope.

### Immunofluorescence

Paraffin-embedded heart sections were deparaffinized in xylene and rehydrated through graded ethanol solutions. Antigen retrieval was performed by heating the sections in citrate buffer (pH 6.0) using a microwave or water bath. After cooling to room temperature, sections were permeabilized with 0.1% Triton X-100 in PBS and blocked with 5% goat serum for 30 minutes. The sections were then incubated overnight at 4°C with primary antibodies or PBS (as negative control). Following washing, Alexa Fluor-conjugated secondary antibodies were applied for 1 hour at room temperature. Finally, sections were mounted using an antifade mounting medium containing DAPI (Beyotime, Cat# P0131-25ml). Confocal imaging was performed using a laser scanning confocal microscope (AX, Nikon, Japan). Fluorescence intensity was quantified using ImageJ software (NIH, USA).

### Transmission electron microscopy (TEM)

Cardiac ultrastructure was examined using transmission electron microscope (HT7700, Hitachi, Japan). Left ventricular tissues were sliced (100 μm) and fixed in 1–2.5% glutaraldehyde in 0.1 mol/L phosphate buffer (pH 7.4) for 15 minutes, washed, and postfixed with 1% osmium tetroxide. Samples were dehydrated, thin sections were stained with uranyl acetate and lead citrate for observation. Images were digitally acquired for analysis.

### RNA Extraction and Reverse Transcription

Upon euthanasia, tissues were immediately frozen in liquid nitrogen. Frozen tissues were homogenized in TRIzol Reagent (Invitrogen, Cat# 15596018) using homogenizer (Shanghai Jinxin Industrial Development, Cat# JXFSTPRP-CL-BSC), Cells were also homogenized directly in TRIzol Reagent. Following homogenization, chloroform (200 μL per 1 mL TRIzol) was added for phase separation. Total RNA was extracted according to the manufacturer’s instructions. RNA was eluted in approximately 20 μL RNase-free water, and concentration was measured using a Nanodrop ND-100 Spectrophotometer. For reverse transcription, 1 μg of total RNA per sample was used with the reverse transcription kit (Yeasen, Catalog #11141ES60) following the manufacturer’s protocol with random primers. The resulting cDNA was diluted 10-fold with RNase-free water prior to quantitative PCR analysis.

### Quantitative PCR

Quantitative PCR was performed using SYBR Green PCR Master Mix (Yeasen, Cat# 11201ES08) in combination with target-specific primers (listed in Supplementary Table S2). Amplification reactions were run on a real-time PCR detection system (Bio-Rad, USA). The thermal cycling conditions were as follows: initial activation at 95°C for 2 min, followed by 40 cycles of denaturation at 95°C for 5 sec and combined annealing/extension at 60°C for 10 sec. Gene expression levels were normalized to 18s as the endogenous control and quantified using the 2^(−ΔΔCt) method. Melting curve analysis was conducted to verify the specificity of all primers.

### Western Blotting

Cellular and myocardial tissue samples were lysed in whole cell lysis buffer supplemented with protease and phosphatase inhibitors, and homogenized using a Shanghai Jingxin homogenizer (Model: JXFSTPRP-CL-BSC). Total protein concentrations were determined using the BCA Protein Assay Kit (Thermo Fisher, Cat# WE325491). Equal amounts of protein (40–60 μg per sample) were mixed with 4× loading buffer and denatured at 37°C or 99°C for 10 min. Samples were separated on 10–12.5% SDS-PAGE gels and electrophoresed at a constant voltage (typically 80–120 V depending on gel size and system used). Proteins were subsequently transferred to PVDF membranes (Millipore, Cat# IPVH00010) using wet transfer at 400mA for 1 hours. After transfer, membranes were blocked for 2 hours at room temperature in TBST buffer (Tris-buffered saline with 0.1% Tween-20) containing 5% bovine serum albumin (BSA; Biosharp, Cat# BS114). Membranes were then incubated overnight at 4°C on a rocker with primary antibodies diluted in blocking buffer. After three 10-minute washes in TBST, membranes were incubated with HRP-conjugated species-matched secondary antibodies (diluted 1:5000) for 1 hour at room temperature. Protein bands were visualized using enhanced chemiluminescence (ECL) reagents and detected on a Fusion FX imaging system (Vilber, France). Densitometric quantification of immunoreactive bands was performed using ImageJ software. Band intensities were normalized to GAPDH or total protein loading controls. Detailed information regarding all primary and secondary antibodies used—including host species, catalog numbers, clonality, and dilution ratios—is provided in Supplementary Table S3

### RNA-seq Analysis

#### 1. RNA Isolation and Library Preparation

Total RNA was extracted using the TRIzol reagent (Invitrogen, CA, USA) according to the manufacturer’s protocol. RNA purity and quantification were evaluated using the NanoDrop 2000 spectrophotometer (Thermo Scientific, USA). RNA integrity was assessed using the Agilent 2100 Bioanalyzer (Agilent Technologies, Santa Clara, CA, USA). Then the libraries were constructed using VAHTS Universal V6 RNA-seq Library Prep Kit according to the manufacturer’s instructions. The transcriptome sequencing and analysis were conducted by OE Biotech Co., Ltd. (Shanghai, China).

#### 2. RNA Sequencing and Differentially Expressed Genes Analysis

The libraries were sequenced on an Illumina Novaseq 6000 platform and 150 bp paired-end reads were generated. About 45 raw reads for each sample were generated. Raw reads of fastq format were firstly processed using fastp and the low quality reads were removed to obtain the clean reads. Then about 40 clean reads for each sample were retained for subsequent analyses. The clean reads were mapped to the reference genome using HISAT2 FPKM of each gene was calculated and the read counts of each gene were obtained by HTSeq-count. PCA analysis were performed using R (v 3.2.0) to evaluate the biological duplication of samples.

Differential expression analysis was performed using the DESeq2. P value < 0.05 and foldchange < 0.58 was set as the threshold for significantly differential expression gene (DEGs). Hierarchical cluster analysis of DEGs was performed using R (v 3.2.0) to demonstrate the expression pattern of genes in different groups and samples. The radar map of top 30 genes was drew to show the expression of up-regulated or down-regulated DEGs using R packet ggradar.

Based on the hypergeometric distribution, GO, KEGG pathway, Reactome and WikiPathways enrichment analysis of DEGs were performed to screen the significant enriched term using R (v 3.2.0), respectively. R (v 3.2.0) was used to draw the column diagram, the chord diagram and bubble diagram of the significant enrichment term.

### Measurement of Succinate Levels

Succinate concentrations in cardiac tissues were measured using a commercial succinate colorimetric assay kit (Elabscience, Cat# E-BC-K902-M), following the manufacturer’s instructions. All reagents were equilibrated to room temperature (25°C) prior to use, and the working solutions, color reagent, and standards were prepared as directed.

Cardiac tissues were homogenized in double-distilled water at a ratio of 1:9 (g/mL). The homogenates were centrifuged at 10,000 × g for 10 minutes at 4°C. A total of 400 μL of the resulting supernatant was transferred to a 10 kDa ultrafiltration tube and centrifuged at 12,000 × g for 15 minutes. The filtrate was collected for analysis.

### Measurement of NAD^+^ Levels and NAD^+^/NADH Ratio

NAD^+^ and NADH levels in heart tissues and AC16 cardiomyocytes were measured using a commercial NAD^+^/NADH Assay Kit (Beyotime, Cat# S0175) according to the manufacturer’s instructions. Standard curves were prepared as directed by the kit protocol. Absorbance was measured using a microplate reader, and NAD^+^ and NADH concentrations were calculated based on the standard curve. All samples were assayed in duplicate. Briefly, approximately 20 mg of heart tissue was homogenized in extraction buffer using the Homogenizer. For cellular assays, about 1×10^6^ cells per sample were washed with cold PBS and pellets were stored at −80°C until analysis. Homogenates were centrifuged at 12,000 × g for 8 minutes at 4°C, and the resulting supernatant was collected for analysis.

### Statistical Analysis

Data are presented as mean ± SEM. Statistical comparisons were performed using unpaired Student’s t test, one-way ANOVA followed by Tukey’s post hoc test, or two-way ANOVA with Bonferroni correction, as appropriate. A P value < 0.05 was considered statistically significant. All analyses were conducted using GraphPad Prism software.

## Results

### Downregulation of the Cardiac Succinate–GPR91 Axis in Heart Failure with Preserved Ejection Fraction

**Figure 1.**
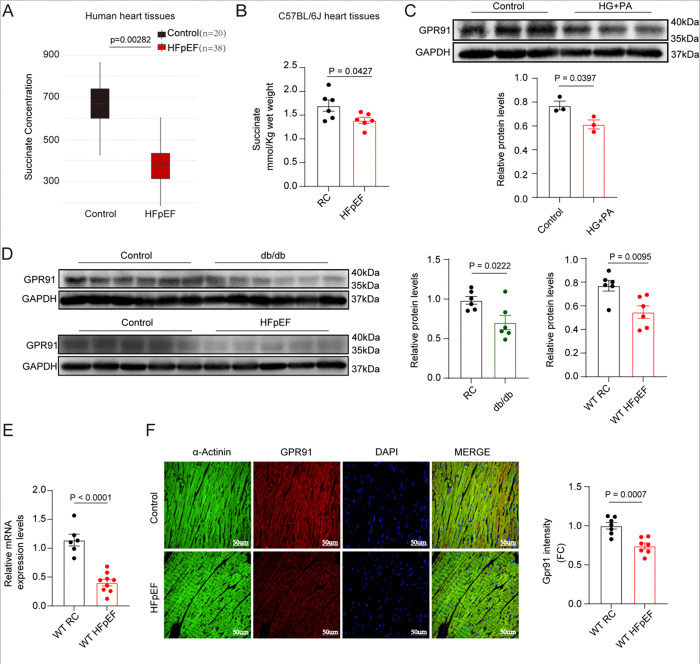
Reduced succinate levels and downregulation of its receptor GPR91 were observed in HFpEF. **(A)**, Succinate levels in cardiac tissue of HFpEF patients, based on targeted metabolomics data from a published study. **(B)**, Succinate levels in cardiac tissue of regular chow (RC) and HFpEF mice, measured using a colorimetric assay. **(C)**, Western blot analysis and quantification for GPR91 expression in AC16 cardiomyocytes under control or HG+PA for 48h. **(D)**, Western blot analysis and quantification for GPR91 expression in the cardiac tissues of control mice compared with db/db or HFpEF mice. **(E)**, The mRNA expression levels of Gpr91 in cardiac tissues of RC and HFpEF mice. **(F)**, Representative images of immunofluorescence staining of GPR91 (Red) in the heart of RC and HFpEF mice. All data are presented as the mean±SEM. P<0.05 are statistically significant and precise values are specified in corresponding figures. Data were analyzed using Kruskal-Wallis test with post hoc Dunn test for multiple comparisons, with adjusted P (Padj) values (Benjamini-Hochberg) provided (A), or analyzed by Student t test (B-F).

To investigate alterations in myocardial succinate levels in both patients with HFpEF and in experimental mouse HFpEF models, we first consulted a recent study published in Circulation (2023), which demonstrated a significant reduction of succinate levels in cardiac tissues from HFpEF patients (Figure 1A). To further validate these findings, we quantified succinate content in cardiac tissues from a “two-hits” (high-fat diet (HFD) combined with L-NAME administration)-induced HFpEF model using a colorimetric assay. Consistent with the human data, myocardial succinate levels were significantly decreased in HFpEF mice compared to controls (Figure 1B). These results indicate that downregulation of cardiac succinate is a conserved metabolic feature of HFpEF across species.

Given the observed reduction in succinate levels in both human and murine HFpEF hearts, we next sought to evaluate the expression of its receptor, GPR91, through which succinate mediates numerous signaling effects. To mimic the metabolic stress conditions typical of HFpEF-such as exposure to high glucose and lipid levels-we treated AC16 cardiomyocytes with high glucose and palmitate (HG+PA) for 48 hours. As expected, GPR91 expression was significantly downregulated following HG+PA treatment compared to the control group (Figure 1C). Concordantly, cardiac tissues from both “two-hit” and db/db induced HFpEF mice exhibited substantially reduced protein levels of GPR91 (Figure 1D), supporting the notion that GPR91 suppression occurs under conditions of metabolic stress and heart failure. Moreover, quantitative PCR analysis revealed a significant decrease in GPR91 mRNA levels in the hearts of HFpEF mice(Figure 1E). This reduction was further confirmed by immunofluorescence staining of heart sections from HFpEF mice, which demonstrated diminished GPR91 expression in HFpEF cardiomyocytes (Figure 1F). Given the established role of succinate as a metabolic signaling molecule and GPR91 as its specific receptor, the coordinated downregulation of both components under metabolic stress suggests an impaired succinate–GPR91 axis may contribute to the pathogenesis and progression of HFpEF.

### Succinate Supplementation Improves Cardiac Diastolic Dysfunction and Metabolic Profile in HFpEF

**Figure 2.**
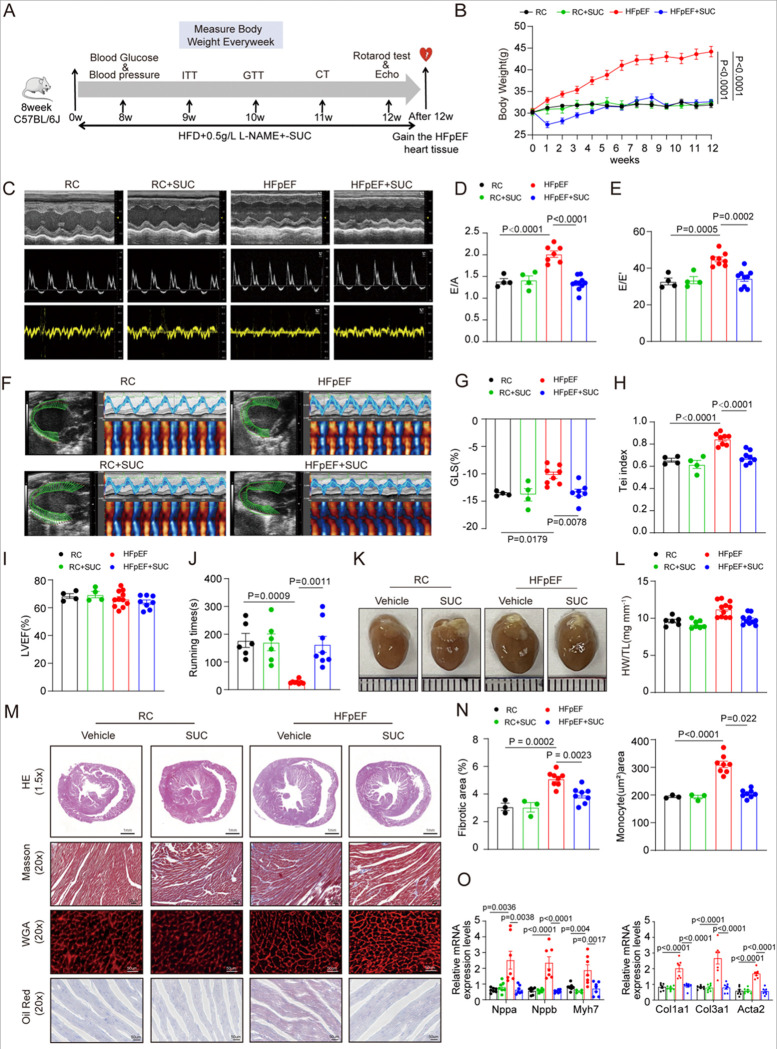
Supplementation with succinate mitigates diastolic dysfunction and metabolic remodeling. C57BL/6J male mice (8-week-old) were fed with regular chow or high-fat diet (HFD)+Nω-nitro-L-arginine methyl ester (l-NAME), and received either control or 1.5% succinate-supplemented drinking water for 12 weeks. **(A)**, Experimental design. **(B)**, Body weight.n=6 in RC and RC+SUC;n=13 in HFpEF and HFpEF+SUC. **(C),** representative images of echocardiography. **(D)**, E/A ratio. **(E)**, E/E′ ratio. **(F)**, Representative image of myocardial strain analysis using VevoStrain. **(G)**, Tei index. **(H)**, Left ventricular global longitudinal strain (GLS). **(I)**, Percentage of LVEF. n=4 in RC and RC+SUC;n=7–10 in HFpEF and HFpEF+SUC. **(J)**, Rotarod-determined running time during the exercise exhaustion test.n=6 in RC and RC+SUC; n=8 in HFpEF and HFpEF+SUC. **(K and L)**, Representative heart images, heart weight (mg) and tibia length(mm) ratio (HW/TL). **(M)**, Representative images of H&E, Masson, WGA and Oil Red-stained sections. **(N)**, Quantification of interstitial fibrosis (%) based on Masson’s trichrome staining, and cardiomyocyte cross-sectional area based on WGA staining. n=3 in RC and RC+SUC;n=8 in HFpEF and HFpEF+SUC. **(O)**, mRNA levels of Nppa, Nppb, and Myh7, Col1a1, Col3a1, and Acta2 in myocardial tissue of mice from different experimental groups.n=6 mice per groups. Each point represents a mouse. All data are presented as the mean±SEM. P<0.05 are statistically significant and precise values are specified in corresponding figures. Data were analyzed by one-way ANOVA (D-O), or by two-way ANOVA (B).

To investigate the role of succinate in HFpEF, C57BL/6J male mice were fed a regular chow or a “two-hit” HFpEF model, with or without 1.5% succinate supplementation in drinking water for 12 weeks (Figure 2A). After 12 weeks of intervention, HFD + L-NAME treatment significantly increased body weight, fat mass, systolic blood pressure, and diastolic blood pressure (Figure 2B; Figure S1A and S1B). As previously reported [35], succinate supplementation effectively inhibited weight gain and fat accumulation. Concurrently, succinate lowered the fasting blood glucose levels, although no significant effect was observed on random glucose measurements (Figure S1C). Consistent with improved metabolic parameters, succinate administration significantly ameliorated glucose intolerance and insulin resistance in HFpEF mice (Figure S1D and S1E). Additionally, succinate supplementation reduced the mass of epididymal white adipose tissue (eWAT), inguinal white adipose tissue (iWAT), and brown adipose tissue (BAT) (Figure S1F and S1G).

Echocardiographic assessment revealed pronounced diastolic dysfunction in HFpEF mice, as evidenced by elevated E/A and E/e’ ratios (Figure 2C through E), indicative of increased left ventricular filling pressure and impaired diastolic function. Speckle-tracking echocardiography revealed a marked reduction in global longitudinal strain (GLS) of the left ventricle in HFpEF mice (Figure 2F and 2G), indicating compromised myocardial deformation. The Tei index was also elevated, reflecting overall cardiac dysfunction (Figure 2H). Importantly, succinate treatment significantly ameliorated cardiac Diastolic dysfunction, underscoring its cardioprotective role.

Notably, neither HFD + L-NAME nor succinate supplementation significantly altered left ventricular ejection fraction (LVEF), consistent with the preserved ejection fraction characteristic of HFpEF (Figure 2I). Exercise capacity, assessed by rotarod test, was notably impaired in HFpEF mice and was improved following succinate administration (Figure 2J). Succinate also reduced heart weight (Figures 2K, 2L) and attenuated myocardial hypertrophy, fibrosis, and lipid deposition observed in HFpEF mice (Figures 2M, 2N). At the molecular level, succinate downregulated the expression of genes related to cardiac hypertrophy and fibrosis (Figure 2O), supporting its role in suppressing maladaptive remodeling. Given that mitochondria represent the major source of succinate and that mitochondrial dysfunction is implicated in diastolic impairment, we performed transmission electron microscopy (TEM) to evaluate ultrastructural changes. Cardiomyocytes from HFpEF mice exhibited mitochondrial swelling, disarray, vacuolization, and cristae disruption—all of which were substantially mitigated by succinate treatment (Figure S1H). These results indicate that succinate helps preserve mitochondrial integrity and attenuates mitochondrial damage in HFpEF. Overall, these findings demonstrate that succinate supplementation attenuates key metabolic, functional, and structural alterations in experimental HFpEF, preserving mitochondrial ultrastructure and supporting cardiac function.

### GPR91 Deficiency Abolishes the Cardioprotective Effects of Succinate in a Mouse Model of HFpEF

**Figure 3.**
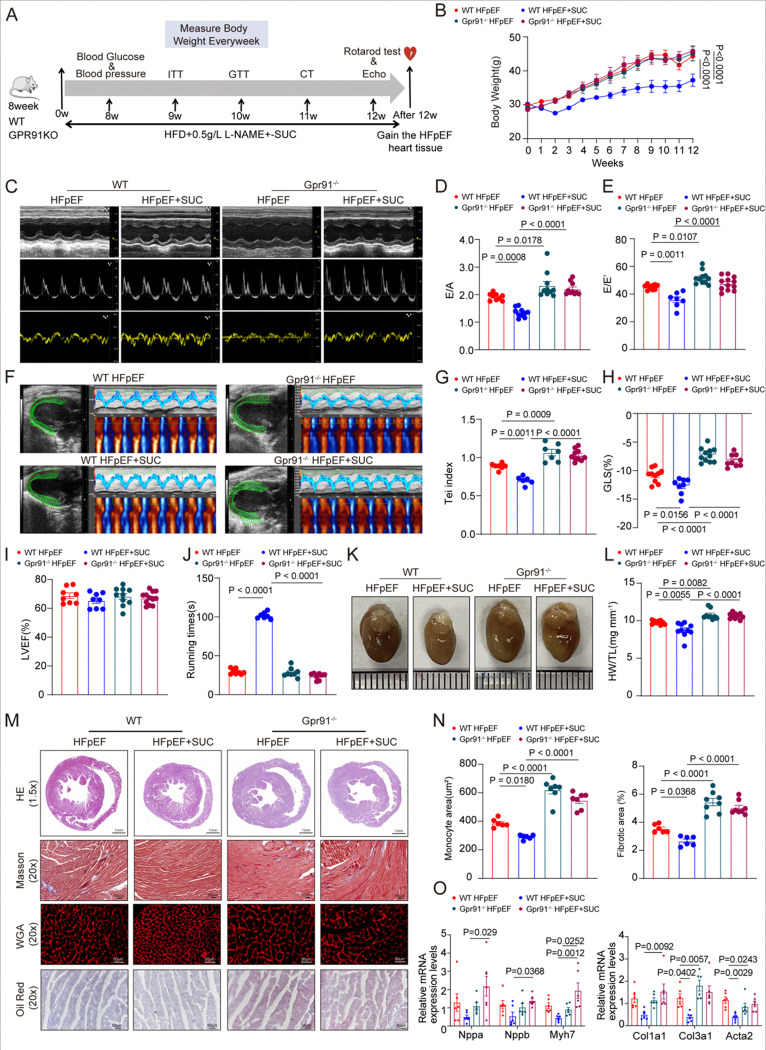
Succinate Fails to Confer Cardiometabolic Protection in GPR91 Knockout HFpEF Mice Global GPR91 knockout (GPR91−/−) and wild-type (WT) C57BL/6J male mice (8-week-old) were fed with either regular chow or HFD+L-NAME, and received either control or 1.5% succinate-supplemented drinking water for 12 weeks. **(A)**, Experimental design. **(B)**, Body weight. n = 7–10. **(C)**, Representative echocardiographic images. **(D)**, E/A ratio. **(E),** E/E’ ratio. **(F)**, Representative myocardial strain analysis using VevoStrain. **(G)**, Tei index. **(H)**, Left ventricular global longitudinal strain (GLS). **(I)**, Left ventricular ejection fraction (LVEF, %). n = 6–12 in each group. **(J)**, Rotarod-determined running time during the exercise exhaustion test. n = 7–8 in each group. **(K and L)**, Representative images of hearts and heart weight/tibia length ratio (HW/TL). n = 9–10 in each group. **(M)**, Representative histological sections stained with H&E, Masson’s trichrome, wheat germ agglutinin (WGA), and Oil Red O. **(N)**, Quantification of interstitial fibrosis (%) based on Masson’s staining, and cardiomyocyte cross-sectional area based on WGA staining. n = 6 in each group. **(O)**, Relative mRNA expression levels of Nppa, Nppb, and Myh7, Col1a1, Col3a1, and Acta2 in cardiac tissue from each group. n = 6 per group. Each data point represents one mouse. Data are expressed as mean ± SEM. P < 0.05 was considered statistically significant, and exact P-values are indicated in the respective figure panels. Data were analyzed using one-way ANOVA (D–O) or two-way ANOVA (B).

Succinate, an intermediate metabolite derived from the mitochondrial tricarboxylic acid cycle, is known to elicit diverse biological responses primarily through its cognate receptor GPR91. Although our previous results demonstrated that exogenous succinate supplementation alleviates cardiac remodeling and diastolic dysfunction in a mouse model of HFpEF (Figure 2), it remained unclear whether these beneficial effects depend on GPR91 signaling. To address this question, we generated global GPR91 knockout (GPR91^−/−^) mice and subjected them to the HFpEF induction protocol with or without succinate administration (Figure 3A). In GPR91-deficient mice, succinate supplementation no longer attenuated weight gain or reduced adipose tissue accumulation (Figure 3B, Figure S2A). The metabolic benefits of succinate were also abolished: fasting blood glucose remained elevated (Figure S2B), and improvements in glucose tolerance and insulin sensitivity observed in wild-type HFpEF mice were absent in GPR91^−/−^ animals (Figures S2C, S2D). Systolic and diastolic blood pressure remained high across all GPR91^−/−^ groups and were unaffected by succinate (Figure S2E). Similarly, succinate failed to reduce fat mass in knockout mice (Figures S2F–S2H).

Echocardiographic assessment revealed exacerbated diastolic dysfunction in GPR91^−/−^ HFpEF mice, indicated by further elevated E/A and E/e’ ratios (Figures 3C–3E), higher Tei index, and reduced GLS compared to wild-type HFpEF controls (Figures 3F–3H). Critically, the improvement in diastolic function previously conferred by succinate was completely absent in GPR91-deficient mice. LVEF remained unchanged, consistent with the HFpEF phenotype (Figure 3I). Exercise capacity, assessed by rotarod test, was severely impaired in GPR91^−/−^ HFpEF mice and unresponsive to succinate treatment (Figure 3J). Macroscopic examination showed pronounced cardiac enlargement in knockout HFpEF mice, accompanied by a significant increase in the heart weight-to-tibia length (HW/TL) ratio (Figures 3K, 3L), suggesting aggravated remodeling that was not mitigated by succinate.

Histological analysis confirmed that GPR91 deletion exacerbated HFpEF-induced myocardial hypertrophy, interstitial fibrosis, and intramyocardial lipid deposition. Succinate treatment provided no protective benefit in the absence of GPR91 (Figures 3M, 3N). Consistently, molecular analysis showed upregulation of hypertrophy- and fibrosis-related genes in GPR91−/− HFpEF hearts, which was not reversed by succinate (Figure 3O). These results underscore that GPR91 deficiency aggravates maladaptive structural changes and clearly indicate that the cardioprotective effects of succinate are mediated through GPR91.

### Succinate Alleviates Cardiac Dysfunction in HFpEF via Cardiomyocyte GPR91 Signaling

Our study has shown that global deletion of GPR91 significantly exacerbates diastolic dysfunction in HFpEF mice, and the cardioprotective effect of succinate is abolished in the absence of GPR91, suggesting that its beneficial actions are dependent on GPR91. However, given that GPR91 is expressed in multiple tissues and cell types, the global knockout model cannot determine its specific role in cardiomyocytes. Existing evidence indicates that various potential pathogenic mechanisms of HFpEF—such as comorbidities, inflammatory responses, and mitochondrial dysfunction—ultimately converge on pathological alterations within cardiomyocytes, positioning them as the final effector cells in HFpEF [36, 37]. Population studies have also demonstrated that changes in succinate levels are predominantly observed in cardiac tissue[12] Furthermore, mitochondrial metabolic reprogramming in cardiomyocytes, including hypertrophy, uncoupling of glycolysis and glucose oxidation, impaired fatty acid uptake and oxidation, and increased ketone body utilization, has been recognized as a key pathophysiological feature of HFpEF.

To further elucidate the cardioprotective mechanism of succinate, we generated cardiomyocyte-specific GPR91 knockout mice (Gpr91^ΔCM^) using a Cre-LoxP system, in order to determine whether the beneficial effects of succinate depend specifically on cardiomyocyte GPR91 signaling. Initial comparison between Gpr91^fl/fl^ and Gpr91^ΔCM^ mice under normal diet conditions revealed no significant differences in glucose tolerance, insulin sensitivity, blood pressure, blood glucose levels, adiposity, or cardiac function (Figures S3A–S3L). Exercise capacity was also comparable between the two groups (Figure S3M), and no differences were observed in heart, lung, or adipose tissue weights (Figures S4A–S4C). However, TEM of cardiac tissue showed increased lipid droplet accumulation and mitochondrial enlargement in Gpr91^ΔCM^ mice (Figure S4D), suggesting underlying alterations in myocardial metabolism.

**Figure 4.**
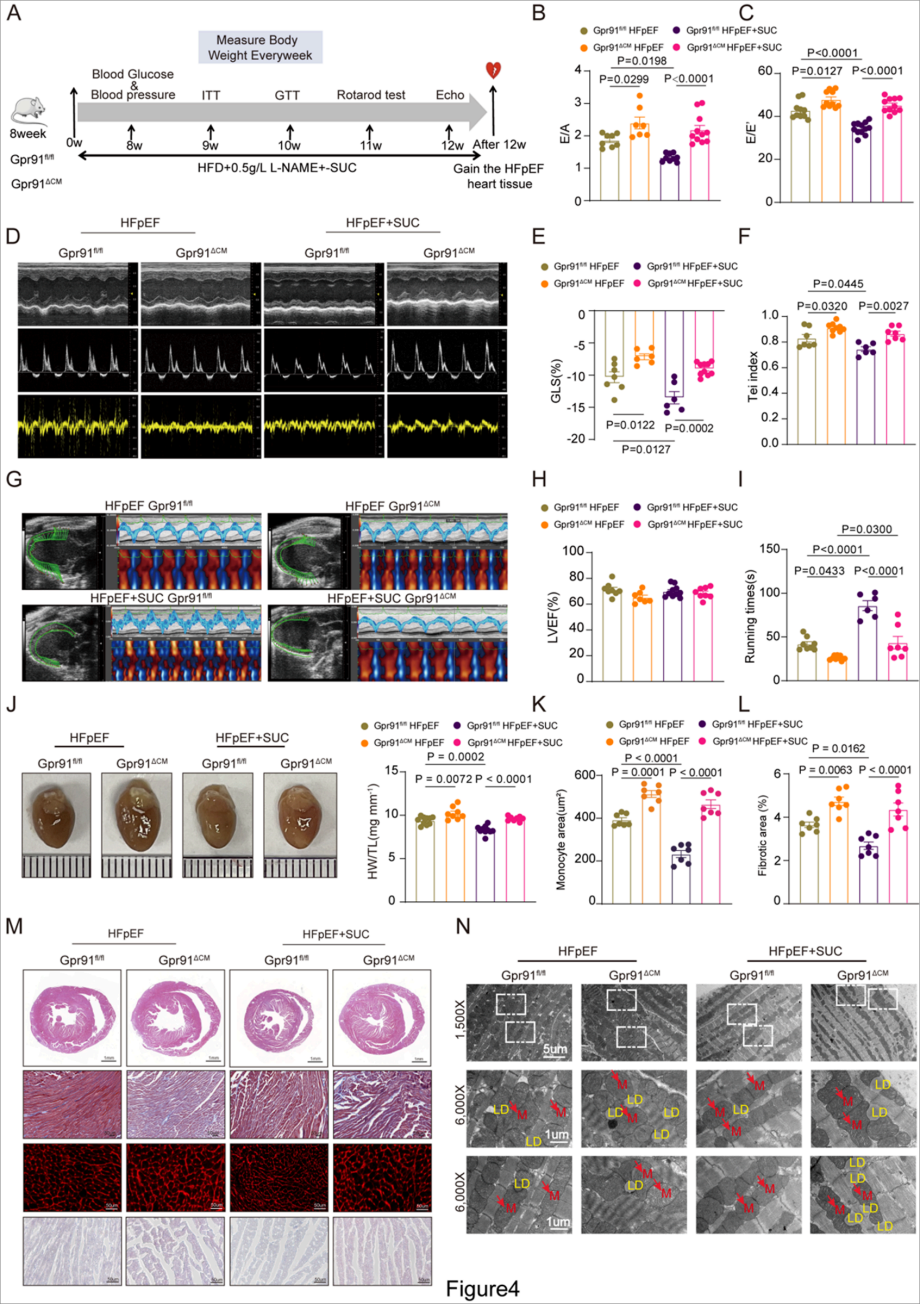
Succinate-Mediated Improvement of Diastolic Dysfunction in HFpEF Depends on Cardiomyocyte GPR91. Cardiomyocyte-specific GPR91 knockout mice and their littermate controls C57BL/6J male mice (8-week-old) were fed with either regular chow or HFD+L-NAME, and received either control or 1.5% succinate-supplemented drinking water for 12 weeks. **(A)**, Experimental design. **(B)**, E/A and**(C),** E/E′ ratio. **(D)**, Representative echocardiographic images. **(E)**, Left ventricular global longitudinal strain (GLS). **(F)**, Tei index. **(G)**, Representative myocardial strain analysis using VevoStrain. **(H)**, Left ventricular ejection fraction (LVEF, %). n = 6–12 in each group. **(I)**, Rotarod-determined running time during the exercise exhaustion test. n = 6–9 in each group. **(J)**, Representative images of hearts and heart weight/tibia length ratio (HW/TL). n = 8–10 in each group. **(K and L)**, Quantification of interstitial fibrosis (%) based on Masson’s staining, and cardiomyocyte cross-sectional area based on WGA staining. n =7 in each group. **(M)**, Representative histological sections stained with H&E, Masson’s trichrome, wheat germ agglutinin (WGA), and Oil Red O. **(N)**, Representative electron microscopy image shows the lipid droplets (yellow) in cardiomyocytes and Mitochondria(red arrow). Data are expressed as mean ± SEM. P < 0.05 was considered statistically significant, and exact P-values are indicated in the respective figure panels. Data were analyzed using one-way ANOVA (B-L).

We then induced HFpEF in both Gpr91^fl/fl^ and Gpr91^ΔCM^ mice through a combination of HFD and L-NAME administration, with or without 1.5% succinate supplementation in drinking water for 12 weeks (Figure 4A). Succinate significantly attenuated weight gain (Figure S5A), improved glucose tolerance and insulin sensitivity (Figures S5B, S5C), and reduced fasting blood glucose in both genotypes (Figure S5D), though no significant effects were observed on random blood glucose or blood pressure (Figures S5D, 5E). Furthermore, succinate reduced overall adiposity, as evidenced by morphological and tissue weight analyses (Figures S5F–S5H). These results indicate that succinate ameliorates systemic metabolic disturbances in HFpEF independently of cardiomyocyte GPR91.

We next assessed whether the cardioprotective effects of succinate require cardiomyocyte GPR91. Echocardiography showed that succinate failed to improve diastolic dysfunction in Gpr91^ΔCM^ mice, with no significant changes in E/A or E/e’ ratios (Figures 4B–4D). Similarly, GLS and Tei index remained unimproved with succinate treatment in knockout mice (Figures 4E–4G). LVEF was preserved across all groups, consistent with the HFpEF phenotype (Figure 4H). Although succinate moderately enhanced exercise capacity-likely due to reduced body weight-this effect was attenuated in Gpr91^ΔCM^ mice (Figure 4I). Gross anatomical and histopathological examinations revealed that cardiomyocyte-specific deletion of Gpr91 exacerbated HFpEF-induced myocardial hypertrophy and interstitial fibrosis, which were not reversed by succinate supplementation (Figures 4J–4M). Accordingly, quantitative PCR showed upregulated expression of hypertrophy- and fibrosis-related genes in Gpr91^ΔCM^ hearts, unaffected by succinate treatment (Figure S5I). Ultrastructural analysis by TEM indicated that Gpr91^ΔCM^ mice exhibited more severe mitochondrial damage, including vacuolization and lipid droplet accumulation, under HFpEF conditions (Figure 4N). Although succinate markedly improved mitochondrial morphology and reduced lipid deposition in Gpr91^fl/fl^ mice, these benefits were absent in Gpr91^ΔCM^ mice. Consistently, quantitative PCR analysis revealed a marked upregulation of hypertrophy-related genes (Nppa, Nppb, and Myh7) and fibrosis-related genes (Col1a1, Col3a1, and Acta2) in the hearts of GPR91^ΔCM^ HFpEF mice, which remained unaffected by succinate treatment (Figure 4O). Collectively, these results demonstrate that the protective effects of succinate on cardiac function and remodeling in HFpEF are specifically mediated through cardiomyocyte GPR91 signaling, independent of its systemic metabolic benefits.

### Cardiomyocyte GPR91-Mediated AMPK Activation by Succinate Restores Metabolic Homeostasis in HFpEF

**Figure 5.**
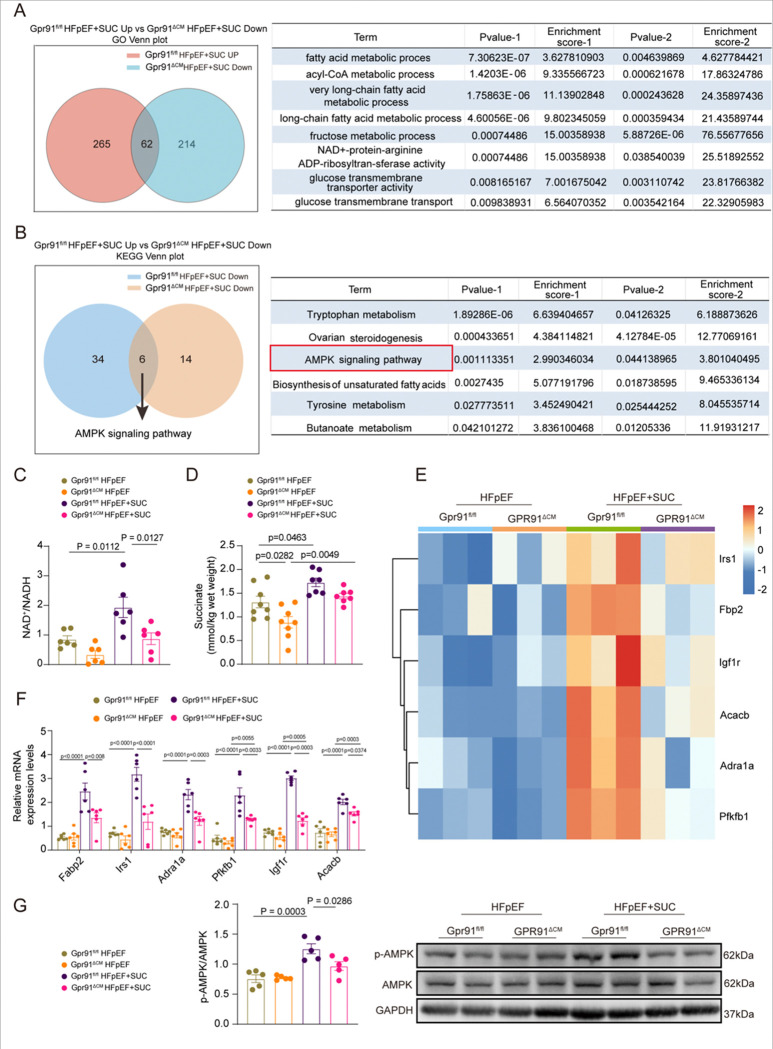
Succinate–GPR91 Activates AMPK to Improve Energy Metabolism in HFpEF Mice **(A),** Venn diagram and table showing shared GO biological processes among groups. **(B)**, Venn diagram and table showing shared KEGG-enriched pathways among groups. **(C)**, Heart NAD^+^/NADH ratio in Gpr91^fl/fl^ and Gpr91^ΔCM^ HFpEF or HFpEF+SUC groups.n=6 in each group. **(D)**, Heart succinate levles in four groups measured by colorimetric assays. **(E)**, Heatmap depicting relative expression of significantly altered AMPK downstream target genes in the hearts. n=3 in each group. **(F)**, qPCR analysis of relative expression levels of AMPK downstream target genes. n=6 in each group. **(G)**, Western blotting analysis of AMPK and phosphorylated AMPK (p-AMPK) levels in heart tissue.n=5 in each group. Data are expressed as mean ± SEM. P < 0.05 was considered statistically significant, and exact P-values are indicated in the respective figure panels. Data were analyzed using one-way ANOVA (D-G).

To further investigate the mechanistic basis of succinate-mediated cardioprotection and its dependence on cardiomyocyte GPR91 signaling, we performed RNA sequencing on cardiac tissues from four experimental groups: Gpr91^fl/fl^ and Gpr91^ΔCM^ HFpEF mice, with or without succinate supplementation. Principal component analysis (PCA) of the RNA-seq data revealed distinct clustering of the different groups, indicating substantial global transcriptional differences (Figure S6A). Volcano plot analysis identified 829 upregulated and 809 downregulated genes in succinate-treated Gpr91^fl/fl^ HFpEF mice, compared to 649 upregulated and 205 downregulated genes in treated Gpr91^ΔCM^ mice (Figure S6B). Gene Ontology (GO) enrichment analysis revealed that succinate upregulated genes related to lipid and glucose metabolism in Gpr91^fl/fl^ mice, whereas these pathways were significantly downregulated in Gpr91^ΔCM^ animals (Figure S7A and S7B), supporting the essential role of cardiomyocyte GPR91 in mediating these metabolic effects. Venn analysis further identified a common set of genes that were upregulated by succinate in Gpr91^fl/fl^ mice but downregulated in Gpr91^ΔCM^ mice, which were predominantly enriched in lipid metabolic processes and NAD^+^ biosynthesis pathways (Figure 5A).

Integrated KEGG pathway analysis of the overlapping gene set highlighted the AMPK signaling pathway as a central regulator of metabolic homeostasis. Succinate robustly activated AMPK signaling in Gpr91^fl/fl^ HFpEF mice, but this response was absent in Gpr91^ΔCM^ littermates (Figure 5B, S8A-B). Given the critical role of AMPK in coordinating energy metabolism, we further validated key glucose and lipid metabolic genes identified by GO analysis using qPCR. Succinate significantly increased their expression in Gpr91^fl/fl^ mice, whereas expression levels remained low or decreased in Gpr91^ΔCM^ mice (Figures S7A, S7B). Consistent with the transcriptomic findings, enzymatic assays showed that succinate supplementation significantly increased cardiac NAD^+^ levels and the NAD^+^/NADH ratio in Gpr91^fl/fl^-but not Gpr91^ΔCM^-mice (Figure 5C, S7C), indicating a GPR91-dependent enhancement of NAD^+^ biosynthesis.

We next examined whether myocardial succinate levels were altered by genotype or treatment. Colorimetric assays revealed reduced endogenous succinate in untreated Gpr91^ΔCM^ HFpEF mice compared to Gpr91^fl/fl^ controls. Exogenous succinate administration restored cardiac succinate content to similar levels in both groups (Figure 5D), suggesting that although GPR91 deletion affects basal succinate abundance, supplemental succinate can achieve comparable tissue concentrations. Nevertheless, the absence of GPR91 prevents subsequent activation of NAD^+^ biosynthetic pathways.

Hierarchical clustering of AMPK downstream targets showed strong upregulation in succinate-treated Gpr91^fl/fl^ mice, with no similar pattern in Gpr91^ΔCM^ animals (Figure 5E). These results were confirmed by qPCR (Figure 5F), underscoring that succinate-induced AMPK activation requires cardiomyocyte GPR91. Finally, western blot analysis demonstrated increased AMPK phosphorylation in succinate-treated Gpr91^fl/fl^ HFpEF mice, which was markedly attenuated in Gpr91^ΔCM^ hearts (Figure 5G). In summary, these data indicate that succinate activates the AMPK signaling pathway in a cardiomyocyte GPR91-dependent manner, leading to improved glucose and lipid metabolism and enhanced NAD^+^ biosynthesis, thereby contributing to the restoration of metabolic homeostasis in HFpEF.

### The Succinate Activates GPR91-AMPK Signaling via Gq to Improve Metabolic Homeostasis and NAD^+^ Biosynthesis

**Figure 6.**
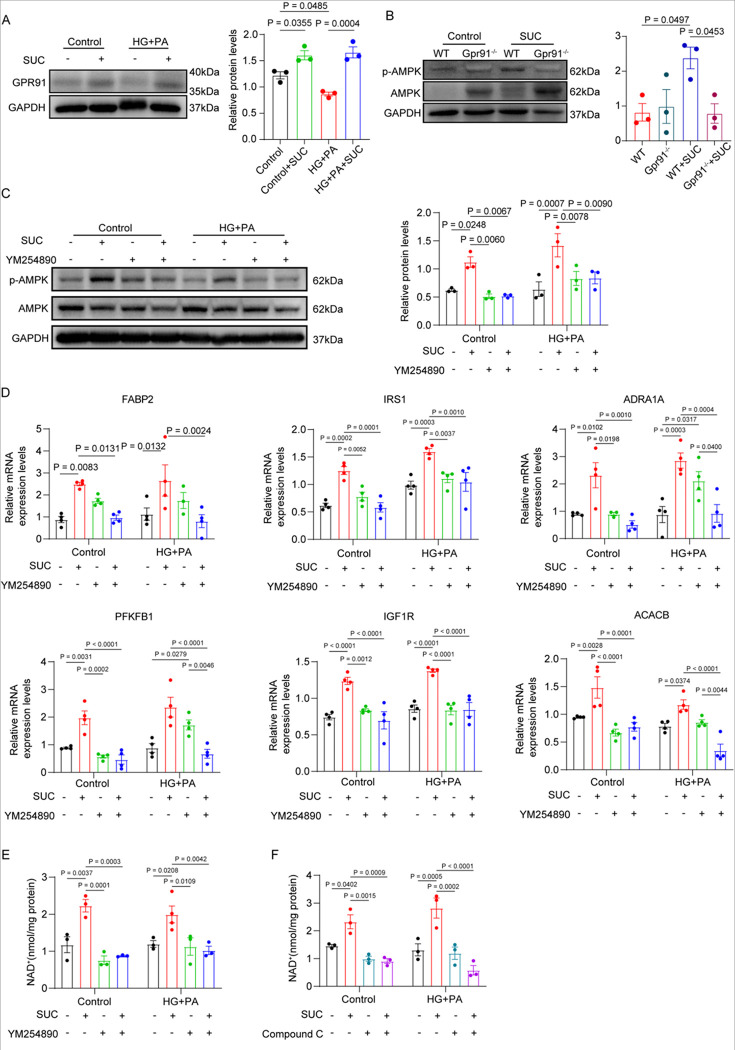
Gq-Dependent AMPK Activation Mediates Succinate–GPR91 Metabolic Effects. AC16 were treated with high glucose(33mM) plus palmitate(200μM) for 48 hours in a sustainable medium to mimic HFpEF-like conditions in vitro. (A), Representative immunoblot images and densitometry analysis of GPR91 in AC16 cells treated with succinate (400μM) under normal or HG+PA conditions. n=3 in each group.(B), Representative immunoblot images and densitometry analysis of p-AMPK/AMPK in primary neonatal ventricular cardiomyocytes isolated from WT and GPR91^−/−^ mice treated with succinate under normal or HG+PA conditions. n=3 in each group (C), AC16 cells were treated with high glucose(33mM) plus palmitate(200μM) for 48 hours, then 1μM Gqi pretreat 1h, succinate treat 5min, Representative blot images of p-AMPK and AMPK. n=3 in each group. (D), qPCR analysis of AMPK downstream target genes in AC16 cells under indicated treatments, showing upregulation by succinate reversed by Gqi. n=4 in each group. (E), Intracellular NAD^+^ levels in AC16 cells treated with succinate and/or Gqi(1μM) in normal and HG+PA environments. (F), NAD^+^ levels following treatment with succinate and/or AMPK inhibitor (Compound C 10μM) in normal and HG+PA environments. n=3 in each group. Data are expressed as mean ± SEM. P < 0.05 was considered statistically significant, and exact P-values are indicated in the respective figure panels. Data were analyzed using one-way ANOVA(A and B) and two-way ANOVA (C-F).

Given that succinate promotes AMPK phosphorylation via cardiomyocyte GPR91 in an HFpEF mouse model, we sought to delineate the upstream mechanisms driving AMPK activation. As a G protein-coupled receptor (GPCR), GPR91 signals through various G proteins, including Gi and Gq [38]. Previous studies demonstrated that GPR91 preferentially couples with Gq, triggering the PLCβ-IP_3_-Ca^2+^ cascade to modulate cellular functions [39]. Increased intracellular Ca^2+^ resulting from Gq activation may promote AMPK phosphorylation through pathways involving calcium/calmodulin-dependent protein kinase kinase β (CaMKKβ) [40]. We therefore hypothesized that succinate activates AMPK via the GPR91-Gq axis to regulate cardiomyocyte metabolism. This hypothesis was tested using both primary cardiomyocytes and the AC16 cell line under conditions mimicking HFpEF. AC16 were exposed to high glucose and palmitate (HG+PA) for 48 hours to establish a metabolic stress model. Western blot analysis showed that succinate increased GPR91 expression under both normal and HG+PA conditions (Figure 6A). In primary neonatal cardiomyocytes from WT and GPR91−/− mice, succinate stimulation significantly enhanced AMPK phosphorylation in WT but not in GPR91−/− cells (Figure 6B), indicating that succinate activates AMPK phosphorylation via cardiomyocyte GPR91. Further experiments in AC16 cells demonstrated that succinate increased AMPK phosphorylation under both normal and HG+PA conditions; however, treatment with a Gq inhibitor significantly attenuated AMPK phosphorylation, and succinate was unable to restore AMPK activation under Gq inhibition (Figure 6C). Consistent with these results, qPCR analysis showed that succinate upregulated AMPK downstream target genes, an effect abolished by Gq inhibition (Figure 6D). These data indicate that succinate activates AMPK in a GPR91- and Gq-dependent manner. We next evaluated the role of this signaling axis in NAD^+^ biosynthesis. Based on preliminary time-course experiments indicating peak NAD^+^ levels at 1 hour after succinate stimulation (Figure S9A), this time point was selected for subsequent assays. Succinate significantly increased intracellular NAD^+^ under both normal and HG+PA conditions, while Gq inhibition suppressed NAD^+^ levels regardless of succinate treatment (Figure 6E, S9B). Furthermore, inhibition of AMPK with Compound C abolished succinate-induced NAD^+^ generation (Figure 6F, S9C). Collectively, these results confirm that succinate activates the GPR91-Gq-AMPK signaling cascade in cardiomyocytes, leading to enhanced NAD^+^ biosynthesis and improved metabolic homeostasis.

### NAM Restores cardiac dysfunction by enhancing NAD^+^ levels in Gpr91^−/−^ HFpEF mice

**Figure 7.**
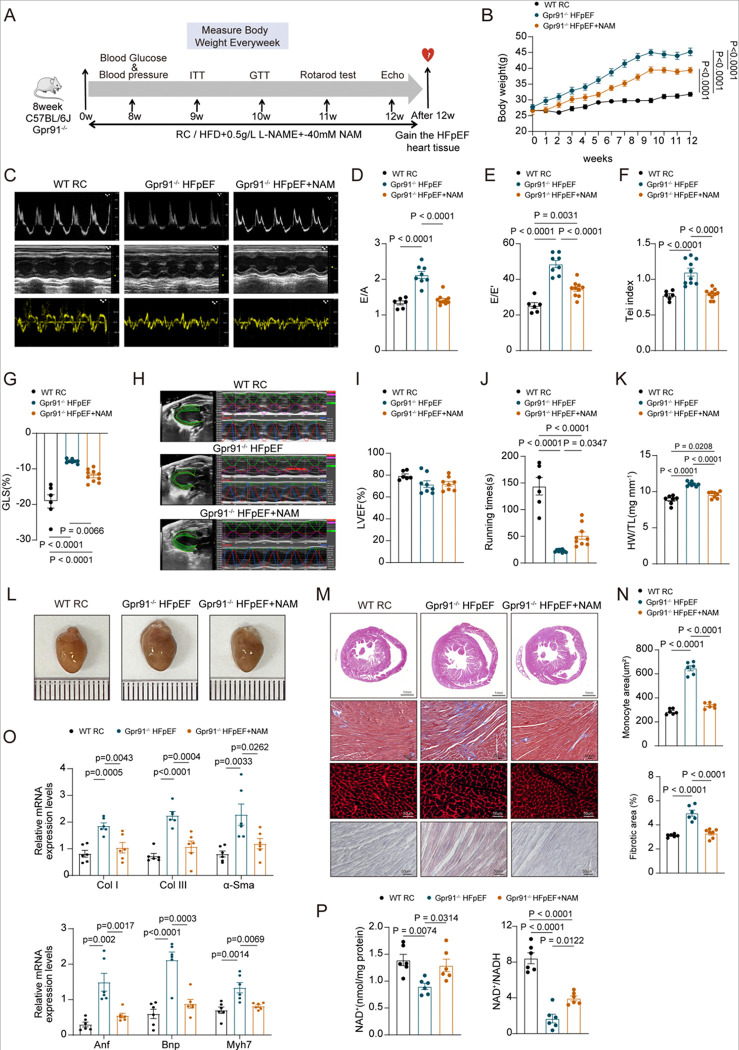
NAM supplementation restores cardiac function and NAD^+^ levels in Gpr91^−/−^HFpEF mice. WT mice were fed with RC diet. Gpr91^−/−^ mice receiving HFD + L-NAME were treated with or without 40mM nicotinamide in drinking water for 12 weeks.**(A)**, Experimental design. **(B)**, Body weight. n = 6–10. **(C)**, Representative echocardiographic images. **(D)**, E/A ratio. **(E),** E/E’ ratio. **(F)**, Representative myocardial strain analysis using VevoStrain. **(G)**, Tei index. **(H)**, Left ventricular global longitudinal strain (GLS). **(I)**, Left ventricular ejection fraction (LVEF, %). n = 6–10 in each group. **(J)**, Rotarod-determined running time during the exercise exhaustion test. n = 6–10 in each group. **(K and L)**, Representative images of hearts and heart weight/tibia length ratio (HW/TL). n =7–8 in each group. **(M)**, Representative histological sections stained with H&E, Masson’s trichrome, wheat germ agglutinin (WGA), and Oil Red O. **(N)**, Quantification of interstitial fibrosis (%) based on Masson’s staining, and cardiomyocyte cross-sectional area based on WGA staining. n = ? in each group. **(O)**, Relative mRNA expression levels of Anf, Bnp, Myh7, Col I, Col III, and α-Sma in cardiac tissue from each group. n = 6 per group. **(P)**, Heart NAD^+^ levels and NAD^+^/NADH ratio in WT RC, Gpr91^−/−^ HFpEF or HFpEF+NAM groups.n=6 in each group. Each data point represents one mouse. Data are expressed as mean ± SEM. P < 0.05 was considered statistically significant, and exact P-values are indicated in the respective figure panels. Data were analyzed using one-way ANOVA (D–P) or two-way ANOVA (B).

Based on the above findings indicating that succinate exerts cardioprotection partly through enhancing NAD^+^ biosynthesis, we hypothesized that NAD^+^ replenishment could rescue cardiac dysfunction even in the absence of GPR91. To test this, we performed a rescue experiment by supplementing Gpr91-deficient HFpEF mice with nicotinamide (NAM), a precursor of NAD^+^, to evaluate whether restoring NAD^+^ levels could compensate for the lack of succinate–GPR91 signaling and ameliorate HFpEF phenotypes. WT mice maintained on a regular chow diet were used as negative controls, while Gpr91^−/−^ mice were subjected to a HFD+L-NAME to induce HFpEF, with or without 40mM NAM supplementation for 12 weeks (Figure 7A). NAM administration markedly attenuated weight gain in Gpr91^−/−^ mice (Figure 7B) and improved glucose tolerance, insulin sensitivity, and fasting blood glucose levels (Figure S10A through S10C). No significant changes were observed in blood pressure (Figure S10D). In addition, NAM supplementation decreased adiposity (Figure S10E through S10G). Echocardiographic analysis further revealed that NAM administration significantly improved diastolic function, as indicated by reduced E/A and E/E’ ratios (Figure 7C through 7E), Meanwhile, a reduction in the Tei index and a significant improvement in GLS were observed (Figure 7F through 7H). LVEF remained preserved (Figure 7I), consistent with a HFpEF phenotype. In addition, rotarod performance was improved by NAM treatment (Figure 7J), indicating enhanced exercise capacity. Gross morphological examination of the heart revealed that NAM treatment attenuated cardiac hypertrophy in GPR91^−/−^ HFpEF mice, as evidenced by a reduced heart weight-to-tibia length ratio(Figure 7K through 7L). HE and Masson staining showed that NAM supplementation alleviated myocardial hypertrophy and interstitial fibrosis (Figure 7M and 7N), which was corroborated by qPCR data showing decreased expression of genes associated with hypertrophy and fibrosis (Figure 7O). NAM supplementation significantly elevated myocardial NAD^+^ levels and restored the NAD^+^/NADH ratio in GPR91^−/−^ mice (Figure 7P). These results demonstrate that restoring NAD^+^ bioavailability alleviates cardiac dysfunction and metabolic abnormalities in Gpr91^−/−^ HFpEF mice, supporting the conclusion that NAD^+^ acts as a crucial downstream mediator of the succinate-GPR91-AMPK axis.

## Discussion

HFpEF is recognized as a metabolic disorder characterized by impaired energy metabolism[41–46]. Yet, how these metabolic perturbations drive cardiac remodeling and diastolic dysfunction remains incompletely understood. In the present study, we identify the succinate–GPR91 axis as a critical mediator linking systemic metabolic cues to cardiomyocyte energetics and function. We show that succinate supplementation ameliorates HFpEF by improving diastolic function and attenuating adverse remodeling, and these benefits are abolished in cardiomyocyte-specific GPR91 knockout mice. Thus, cardiomyocyte GPR91 emerges as an essential receptor coupling extracellular succinate to myocardial metabolic adaptation. Mechanistically, succinate–GPR91 signaling promotes metabolic reprogramming within cardiomyocytes by activating AMPK and enhancing NAD^+^ biosynthesis, thereby restoring energy homeostasis and restoring diastolic function. Notably, even in the absence of GPR91, supplementation with the NAD^+^ precursor NAM similarly improves HFpEF, indicating that NAD^+^ generation functions as a key downstream effector of succinate signaling and represents a potential therapeutic target. Collectively, these findings establish a mechanistic framework in which succinate supplementation activates cardiomyocyte GPR91 to drive AMPK-dependent NAD^+^ biosynthesis and metabolic reprogramming, thereby restoring energy balance and diastolic function in HFpEF, and identify both the succinate–GPR91 axis and NAD^+^ generation as promising therapeutic avenues.

Succinate was viewed as a metabolite with context-dependent and sometimes paradoxical effects. Succinate promotes Ractive oxygen species(ROS) generation and cardiac injury during ischemia while driving vascular inflammation and aneurysm progression[16, 47–52], yet conferring metabolic benefits in the liver, adipose, and pancreas through GPR91-dependent signaling [35, 53–57]. Given that HFpEF represents a systemic metabolic disorder characterized by obesity, insulin resistance, and chronic inflammation, our findings suggest that succinate supplementation simultaneously alleviates systemic metabolic stress and directly reprograms cardiomyocyte metabolism to restore diastolic function during HFpEF.

Moreover, our study highlights cardiomyocyte GPR91 as a critical node that integrates systemic metabolic cues with local myocardial responses.

Mechanistically, previous studies have shown that GPR91 primarily couples with Gq proteins in various cell types, activating the PLCβ–IP_3_–Ca^2+^ signaling cascade to regulate cellular functions[58]. Notably, Gq-induced elevations in intracellular Ca^2+^ may contribute to the activation of AMPK via CaMKKβ-mediated pathways[40]. Activated AMPK, in turn, upregulates NAMPT and enhances NAD^+^ biosynthesis[59], thereby restoring energy homeostasis. In the present study, we links extracellular succinate to intracellular metabolic reprogramming and diastolic improvement by activating GPR91-Gq-AMPK-NAD^+^ axis. This novel pathway highlights a molecular mechanism through which succinate supplementation restores cardiomyocyte metabolism and mitigates HFpEF progression. Interestingly, our study also demonstrates that supplementation with the NAD^+^ precursor NAM rescued HFpEF even in the absence of cardiomyocyte GPR91, underscoring NAD^+^ as a downstream effector and a potential therapeutic target.

These findings advance several conceptual insights. First, they establish cardiomyocyte GPR91 as a metabolic sensor that integrates systemic succinate availability with myocardial energetic adaptation. Second, they reveal a novel signaling mechanism in which GPR91 engages AMPK to drive NAD^+^ biosynthesis, directly connecting extracellular metabolites to cardiomyocyte metabolic resilience. Third, they suggest that NAD^+^ replenishment can bypass upstream receptor deficiency, highlighting translational opportunities for metabolic therapy in HFpEF.

Several limitations should be acknowledged. While our mouse model recapitulates cardinal features of human HFpEF, interspecies differences may limit generalizability. Moreover, the role of succinate–GPR91 signaling in non-cardiomyocyte populations warrants further investigation. Finally, the long-term efficacy and safety of succinate or NAD^+^ precursors in patients with multiple comorbidities remain to be determined.

## Conclusion

Our findings identify the succinate–GPR91–AMPK–NAD^+^ axis as a novel metabolic pathway that restores cardiomyocyte energetics and improves diastolic function in HFpEF. By establishing cardiomyocyte GPR91 as a key metabolic sensor and NAD^+^ as a critical downstream effector, this work highlights promising therapeutic strategies that target metabolic remodeling to mitigate HFpEF progression.

## Supplementary Material

Supplementary Files

This is a list of supplementary files associated with this preprint. Click to download.

• Additionalfile1.docx

• Graphicalabstract.docx

## Data Availability

The RNA-seq data supporting the findings of this study are not yet publicly available but can be obtained from the corresponding author upon reasonable request. The data will be deposited in a public repository (NCBI GEO) upon acceptance of the manuscript, and the accession number and link will be provided at that time.

## Research Insights

### What is currently known about this topic?

HFpEF is a systemic metabolic disease; cardiomyocyte dysfunction and mitochondrial impairment are hallmarks.

Succinate, a key intermediate of the tricarboxylic acid (TCA) cycle, is significantly reduced in the myocardium of HFpEF.

Succinate serves as an endogenous ligand for the G-protein–coupled receptor GPR91, linking metabolic status to cellular signaling.

### What is the key research question?

Does succinate–GPR91 signaling in cardiomyocytes regulate metabolic reprogramming and thereby influence the development and progression of HFpEF?

### What is new?

Succinate supplementation ameliorates HFpEF by improving myocardial metabolism, diastolic function, and adverse remodeling through cardiomyocyte GPR91. Succinate–GPR91 signaling engages the Gq–AMPK–NAD? biosynthetic pathway, thereby promoting cardiomyocyte metabolic reprogramming and restoring energy homeostasis.

### How might this study influence clinical practice?

Targeting the succinate–GPR91 signaling pathway may represent a novel therapeutic approach to restore myocardial energetics and improve diastolic function in HFpEF.

Supplementation with the NAD? precursor nicotinamide (NAM) can bypass GPR91 deficiency, highlighting NAD? replenishment as an alternative or adjunctive strategy for treating HFpEF.

## Abbreviations

AMPK: AMP-activated protein kinase
BAT: Brown Adipose Tissue
CaMKKβ: Calmodulin-Dependent Protein Kinase Kinase β
ECL: Enhanced Chemiluminescence
eWAT: Epididymal White Adipose Tissue
FS: Fractional Shortening
GLS: Global Longitudinal Strain
GO enrichment: Gene Ontology Enrichment
GPCR: G protein-coupled receptor
GPR91: G Protein-Coupled Receptor 91
H&E: Hematoxylin And Eosin
HFD: High-Fat Diet
HFpEF: Heart failure with preserved ejection fraction
HG+PA: High Glucose And Palmitate
iWAT: Inguinal White Adipose Tissue
L-NAME: Nω-Nitro-L-arginine Methyl Ester Hydrochloride
LV: Left Ventricular
LVEF: Left Ventricular Ejection Fraction
NAD+: Nicotinamide Adenine Dinucleotide
NAM: Nicotinamide
PCA: Principal Component Analysis
PWD: Pulsed-Wave Doppler
RAS: Renin-Angiotensin System
ROS: Ractive Oxygen Species
TCA: Tricarboxylic Acid
TDI: Tissue Doppler Imaging
TEM: Transmission Electron Microscopy
TEM: Transmission Electron Microscopy
WGA: Wheat Germ Agglutinin
WT: Wild-Type
